# First line Immunotherapy for Non-Small Cell Lung Cancer

**DOI:** 10.3390/ph13110373

**Published:** 2020-11-08

**Authors:** Nicola J. Nasser, Miguel Gorenberg, Abed Agbarya

**Affiliations:** 1 Department of Radiation Oncology, University of Maryland School of Medicine, Maryland Proton Treatment Center, Baltimore, MD 21201, USA; 2 Department of Nuclear Medicine, Bnai Zion Medical Center; the Ruth & Bruce Rappaport Faculty of Medicine, Technion-Israel Institute of Technology, Haifa 31048, Israel; miguel.gorenberg@b-zion.org.il; 3 Institute of Oncology, Bnai Zion Medical Center, Haifa 31048, Israel; abed.agbarya@b-zion.org.il

**Keywords:** lung cancer, monoclonal recombinant antibodies, chemotherapy, immune checkpoints inhibitors, programmed death receptor, CTLA-4

## Abstract

Immunotherapy for non-small cell lung cancer (NSCLC) is incorporated increasingly in first line treatments protocols. Multiple phase 3 studies have tested different medications targeting programmed death receptor 1 (PD-1), programmed death-ligand 1 (PD-L1), cytotoxic T-lymphocyte-associated protein 4 (CTLA-4), with or without chemotherapy. The inclusion criteria differ between the various clinical trials, including the cut-off levels of PD-L1 expression on tumor cells, and the tumor histology (squamous or non-squamous). Patients with tumor expression levels of PD-L1 ≥ 50% are candidates for treatment with single agent Pembrolizumab or Atezolizumab. Patients with PD-L1 < 50% are candidates for immunotherapy with pembrolizumab as a single agent if PL-1 > 1%; immunotherapy doublet, Nivolumab and Ipilimumab, or single agent immunotherapy combined with chemotherapy. Here we review phase 3 clinical trials utilizing immunotherapy in the first line for treatment of NSCLC, including Pembrolizumab in KEYNOTE-024, KEYNOTE-042, KEYNOTE-189 and KEYNOTE-407; Nivolumab and Ipilimumab in CHECKMATE-227 and CHECKMATE 9LA; and Atezolizumab in IMpower110, IMpower130 and IMpower150.

## 1. Introduction

Lung cancer is the leading cancer killer in both men and women in the Unites States, with over 135,000 deaths expected during 2020 [1]. Small cell lung cancer accounts for 15%, and non-small cell lung cancer (NSCLC) for 85% of lung cancer cases [2]. Immunotherapy for NSCLC uses monoclonal antibodies that targets immune system T cells or ligands on the tumors cells, and results in enhanced immune system mediated tumor-cell-kill [3].

**Figure 1:**
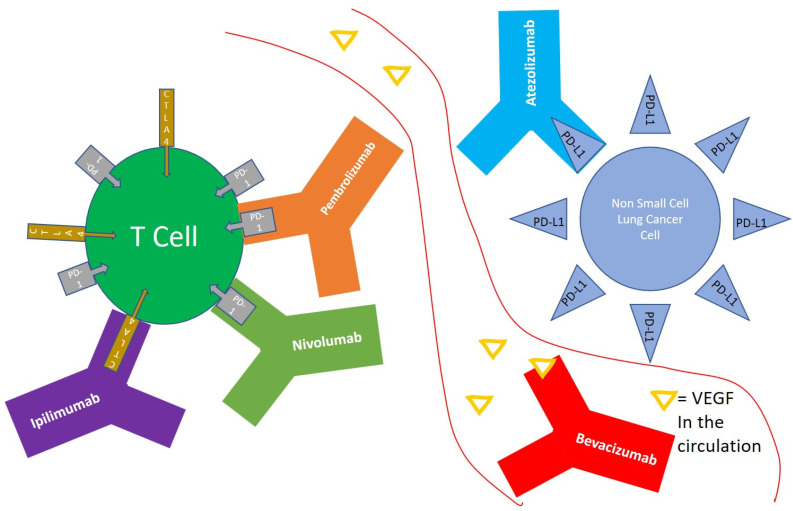
Non-small cell lung cancer (NSCLC) cells expressing programmed death-ligand 1 (PD-L1) could interact with programmed death receptor 1 (PD-1) expressed on the surface of T cells, and result in decreased tumor cell kill by the immune system. Atezolizumab is an anti PD-L1 monoclonal antibody. Nivolumab and Pembrolizumab are anti PD-1 monoclonal antibodies. Ipilimumab is a monoclonal antibody that targets Cytotoxic T-lymphocyte-associated protein 4 (CTLA-4) on the surface of T cells. Bevacizumab is a monoclonal antibody that targets Vascular Endothelial Growth Factor (VEGF) in the circulation and functions as an angiogenesis inhibitor.

T-cells primed to respond to tumor cell, are exposed continuously to tumor antigens during active malignancy, which may result in upregulation of multiple inhibitory receptors, culminating in less action against the tumor cell, in what is known as T-cell exhaustion [4]. T-cell exhaustion could be overcome by modulating the inhibitory pathways that are upregulated during this process [4]. Programmed death receptor 1 (PD-1) is expressed on the surface of T cells, and functions as an immune checkpoint that suppresses autoimmunity through multiple mechanisms [5], and is actually a marker of T-cell exhaustion. NSCLC tumor cells expressing programmed death-ligand 1 (PD-L1) could attach to PD-1 receptor on T cells, and result in decreased tumor cell kill by the immune system [6]. Pembrolizumab [7] and Nivolumab [8] are monoclonal antibodies that target PD-1 on T cells, and shield it from activation by tumors expressing PD-L1 (Figure 1), and thus results in enhanced immune activity. Atezolizumab is a monoclonal antibody that target PD-L1 on tumor cells, prevent it from activating PD-1 on T cells, which results in less suppression of T cell function [9] (Figure 1).

Cytotoxic T-lymphocyte-associated protein 4 (CTLA-4) is a receptor on T cells that functions as an immune checkpoint that downregulates immune responses [10]. Ipilimumab is a monoclonal antibody that targets CTLA-4 and inhibits its activation and thus stimulates the immune system [11] (Figure 1). Dual blockade CTLA-4 and PD-1 therapy was shown to have enhanced efficacy of tumor cell kill in multiple preclinical and clinical trials [12,13], though with an increase in immune related adverse events with these combinations. 

Here we will focus mainly on the role of immunotherapy in the first line treatment of NSCLC. This review will not get into details regarding the treatment of patients with driver mutations, such as anaplastic lymphoma kinase (ALK)-EML4 gene translocation [14] or mutations in the epidermal growth factor receptor (EGFR) [15] as they were excluded from most phase 3 clinical trials incorporating immunotherapy in the first line [16,17,18,19]. 

## 2. Monoclonal Antibodies Targeting Immune Checkpoints

Immune checkpoints refer to inhibitory pathways incorporated in the immune system that are crucial for maintaining self-tolerance [20], and are negative regulators of T-cell immune function. Antibodies targeting immune checkpoints results in enhanced immune mediated tumor cell kill (Table 1).

### 2.1. Nivolumab

Nivolumab is a fully human IgG4 antibody targeting PD-1. Nivolumab is provided intravenously, with a mean half-life of 25 days [21]. No dose adjustment is recommended in patients with renal failure, and mild or moderate hepatic impairment [21]. Most common adverse reactions in patients treated with Nivolumab as a single agent are fatigue, rash, musculoskeletal pain, pruritus, diarrhea, nausea, asthenia, cough, dyspnea, constipation, decreased appetite, back pain, arthralgia, upper respiratory tract infection, pyrexia, headache, abdominal pain, and vomiting [21,22]. Immune-mediated side effects were reported and includes pneumonitis, thyroiditis which could manifest as hypothyroidism and hyperthyroidism [23,24,25,26], colitis [27], hepatitis [28], and nephritis [29]. 

### 2.2. Pembrolizumab

Pembrolizumab is a humanized IgG4 antibody targeting PD-1. Pembrolizumab is provided intravenously, with a mean half-life of 22 days [30]. No dose adjustment is recommended in patients with renal failure, or mild hepatic impairment [30]. Most common adverse reactions in patients treated with Pembrolizumab as a single agent are fatigue, musculoskeletal pain, decreased appetite, pruritus, diarrhea, nausea, rash, pyrexia, cough, dyspnea, constipation, and abdominal pain [30,31,32,33]. Immune-mediated side effects were reported and includes pneumonitis [34], colitis [35,36], hepatitis [37,38], adrenal insufficiency [39,40], hypophysitis [39], hyperthyroidism [41] and hypothyroidism [42,43], type 1 diabetes mellitus [44,45], and nephritis [46,47]. 

### 2.3. Atezolizumab

Atezolizumab is a humanized IgG1 antibody targeting PD-L1. Atezolizumab is provided intravenously, with a half-life of 27 days. Mild or moderate renal failure (estimated glomerular filtration rate (eGFR) ≥ 30 mL/min/1.73 m^2^) and mild to moderate liver failure (bilirubin < 3× upper limit of normal and any aspartate transaminase levels) had no clinically significant effect on the systemic exposure of atezolizumab [48]. The most common adverse reactions in patients treated with Atezolizumab as a single agent are fatigue, nausea, cough, dyspnea, and decreased appetite [48]. Immune-mediated side effects were reported and includes pneumonitis [49,50,51], hepatitis [52,53], colitis [53], hypophysitis [54], thyroid disorders [55], adrenal insufficiency [56], and type 1 diabetes mellitus [57].

### 2.4. Ipilimumab

Ipilimumab is a fully human IgG1 kappa antibody targeting CTLA-4. Ipilimumab is provided intravenously, with a half-life of 15.4 days [58]. The following factors had no clinically important effect on the clearance of ipilimumab: Age, sex, performance status, renal impairment (glomerular filtration rate ≥15 mL/min/1.73 m^2^), or mild hepatic impairment (total bilirubin >1 to 1.5 times the upper limit of normal or aspartate transaminase levels > upper limit of normal) [58]. The most common adverse reactions with Ipilimumab as a single agent are fatigue, diarrhea, pruritus, rash, colitis, nausea, vomiting, headache, weight loss, pyrexia, decreased appetite, and insomnia [58]. Immune-mediated adverse reactions to Ipilimumab were reported and includes pneumonitis [59], colitis [60,61], nephritis [62,63,64,65], myocarditis [57]; endocrinopathies, including thyroid disorders [66], adrenal insufficiency [67], type 1 diabetes mellitus [68,69], and hypophysitis/hypopituitarism [58]. 

### 2.5. Durvalumab

Durvalumab is a fully human IgG1 kappa antibody targeting PD-L1. Durvalumab is provided intravenously, with a half-life of 18 days [70]. Mild (creatinine clearance 60 to 89 mL/min) or moderate renal impairment (creatinine clearance 30 to 59 mL/min), and mild hepatic impairment (bilirubin ≤ upper limit of normal and aspartate transaminase levels > upper limit of normal or bilirubin > 1 to 1.5 x upper limit of normal and any aspartate transaminase levels) had no clinically significant effect on the pharmacokinetics of durvalumab [70]. The most common adverse reactions with Durvalumab as a single agent are fatigue, constipation, rash, nausea, dyspnea, swelling in the arms and legs, and decreased appetite. Immune-mediated adverse reactions to Durvalumab which were reported include pneumonitis [71,72], hepatitis [71,73], colitis [74], nephritis [74], dermatologic reactions [75,76], and endocrinopathies, including thyroiditis [77,78], adrenal insufficiency [77], and type 1 diabetes mellitus [77,78,79].

## 3. Chemotherapeutic Agents Used for Treatment of NSCLC Together with Immunotherapy

### 3.1. Platinum Based Chemotherapeutic Agents

Chemotherapy for NSCLC usually includes combination of two drugs (chemotherapy doublet), with one of the agents is cisplatin or carboplatin. In 2002, Schiller at al. published in the New England Journal of Medicine, a study that compared four chemotherapy regimens for advanced NSCLC, cisplatin and paclitaxel, cisplatin and gemcitabine, cisplatin and docetaxel, and carboplatin and paclitaxel [80]. The study was well powered, with 1207 patients enrolled. None of the four chemotherapy regimens offered a significant advantage over the others [80]. In 2008, Scagliotti et al. [81] published in the Journal of Clinical Oncology a study that compared cisplatin plus gemcitabine with cisplatin plus pemetrexed in chemotherapy-naive patients with advanced NSCLC [81]. Overall survival was statistically superior for cisplatin/pemetrexed versus cisplatin/gemcitabine in patients with adenocarcinoma and large-cell carcinoma histology, while patients with squamous cell histology had a significant improvement in survival with cisplatin/gemcitabine versus cisplatin/pemetrexed [81]. These two studies made significant impact on the chemotherapy choices for treating NSCLC, with cisplatin or carboplatin as the backbone of all the chemotherapy treatments protocols. Pemetrexed is provided with platinum-based chemotherapy to patients with nonsquamous NSCLC. Gemcitabine is provided with platinum-based drug to patients with squamous NSCLC.

#### 3.1.1. Cisplatin

Cisplatin is composed of a central atom of platinum with two chloride atoms and two ammonia molecules attached to it in the cis position. Cisplatin is provided intravenously and exerts its cytotoxic effects through binding to the deoxyribonucleic acid (DNA) strands, making inter- and intra- strands cross-links, which results in disruption of transcription and translation of DNA. Nephrotoxicity is the dose limiting toxicity of cisplatin, which results mainly from proximal tubular injury [82]. Other side effects of cisplatin include nausea and vomiting which usually necessitate premedication with antiemetic medications before cisplatin infusion, ototoxicity which could manifest in varying levels of hearing loss, peripheral neuropathy, and myelosuppression. 

#### 3.1.2. Carboplatin

Carboplatin, like cisplatin is composed of a central atom of platinum and two ammonia molecules, but the two chloride atoms are substituted by a cyclobutanedicarboxylate moiety. Carboplatin is provided intravenously and exerts its cytotoxic effects through inter- and intra- DNA strands cross-links, which results in disruption of transcription and translation of DNA. Bone marrow suppression is the dose-limiting toxicity of carboplatin. Carboplatin is much less nephrotoxic compared to cisplatin and is used as an alternative to cisplatin for patients with preexisting renal failure. 

### 3.2. Taxanes

Taxanes are microtubule-stabilizing drugs which induces mitotic arrest at the G2/M transition phase of the cell cycle, resulting in cell death. Paclitaxel was isolated from bark extract of the Pacific yew tree. Docetaxel is a semisynthetic taxane and nab-paclitaxel is a nanoparticle albumin-bound paclitaxel. 

#### 3.2.1. Paclitaxel

Paclitaxel binds to tubulin and stabilizes the microtubules which leads to inhibition of cell division. Paclitaxel is provided intravenously, with a dose limiting toxicity of peripheral neuropathy. Peripheral sensory neuropathy presents with numbness and tingling in a stocking-and-glove distribution [83], which may disturb daily function of the patients. Hematologic toxicity include anemia, neutropenia, and less frequently thrombocytopenia.

#### 3.2.2. Docetaxel

Docetaxel is provided intravenously, and acts in similar manner to paclitaxel. Docetaxel binds to tubulin, the protein component of the microtubules, and inhibits its disassembly, which results in disruption of mitosis and cell death. Docetaxel appears twice as active as paclitaxel in microtubules depolymerization inhibition [84]. Some clinical studies show that Docetaxel seems to be more potent compared with Paclitaxel, especially for treatment of breast cancer patients [85,86]. Hematological toxicities are the dose limiting toxicity of Docetaxel with neutropenia and anemia. Other side effects include alopecia, stomatitis, diarrhea, nausea, vomiting, fluid retention, onycholysis, and skin toxicity [87,88]. 

#### 3.2.3. Nanoparticle Albumin-Bound Paclitaxel

Nanoparticle albumin-bound (nab) paclitaxel is an Albumin bound with high affinity to the hydrophobic molecules of paclitaxel, which results in higher accumulation of the cytotoxic drug in tumors. A recent meta-analysis showed that when compared to Paclitaxel, nab-paclitaxel has significant beneficial effects in terms of overall response rate, progression free survival, and overall survival [89]. Side effects of nab-paclitaxel include anemia, neutropenia, alopecia, and peripheral neuropathy [90]. 

### 3.3. Gemcitabine

Gemcitabine is structurally similar to cytarabine and functions as a pyrimidine analog, and blocks the progression of cells through the G1/S-phase [91]. Gemcitabine is metabolized by nucleoside kinases to Gemcitabine diphosphate and Gemcitabine triphosphate. Gemcitabine diphosphate inhibits ribonucleotide reductase, resulting in reductions in deoxynucleotide concentrations, including deoxycytidine triphosphate. Gemcitabine triphosphate competes with deoxycytidine triphosphate for incorporation into DNA [91]. Side effects of Gemcitabine includes myelosuppression manifested by neutropenia, thrombocytopenia, and anemia [91,92]; pulmonary toxicity, including interstitial pneumonitis, pulmonary fibrosis, pulmonary edema, and adult respiratory distress syndrome [91,93,94]; capillary leak syndrome [95,96,97]; and posterior reversible encephalopathy syndrome [98,99].

### 3.4. Pemetrexed

Pemetrexed functions as an antimetabolite. Pemetrexed inhibits thymidylate synthase, dihydrofolate reductase and glycinamide ribonucleotide formyltransferase. Pemetrexed induces cell cycle arrest in the G1/S phase. Side effects of Pemetrexed includes myelosuppression [100], renal failure [100], bullous and exfoliative skin toxicity [100,101,102], diarrhea, nausea, and vomiting [100]. 

## 4. Phase 3 Randomized Controlled Trials that Includes Immunotherapy for NSCLC

**Figure 2:**
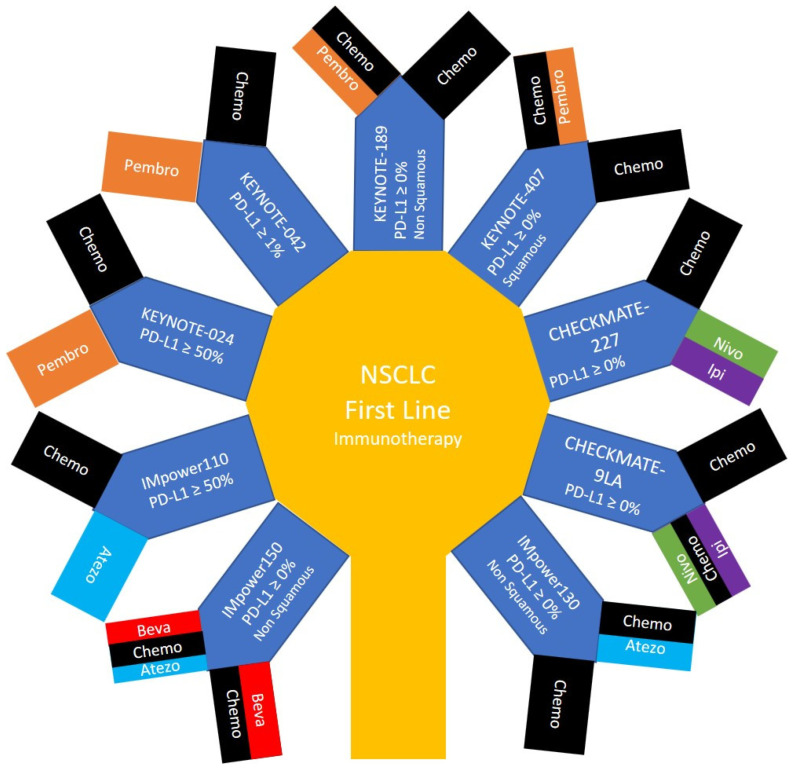
The main treatment arms of phase 3 clinical trials providing immunotherapy in the first line for patients with non-small cell lung cancer.

**Figure 3:**
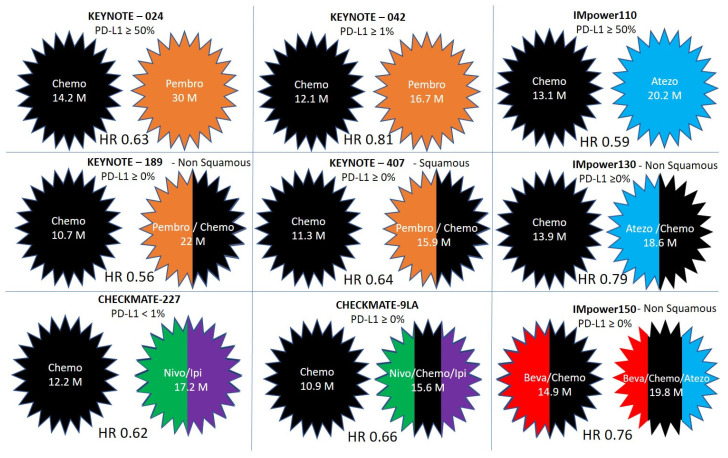
Comparison of over survival and hazard ratios (HR) in clinical trials incorporating immunotherapy in the first line for patients with non-small cell lung cancer. The treatment arms without and with immunotherapy are compared in KEYNOTE-024, KEYNOTE-042, KEYNOTE-189, KEYNOTE-407, CHECKMATE-227, CHECKMATE 9LA, IMpower110, IMpower130, and IMpower150.

Phase 3 randomized controlled trials that compare immunotherapy as a single modality or in combination with other systemic therapies, to the standard of care that was before the publication of these trials are described in Figure 2 and Figure 3. These trials included Pembrolizumab in KEYNOTE-024, KEYNOTE-042, KEYNOTE-189 and KEYNOTE-407; Nivolumab and Ipilimumab in CHECKMATE-227 and CHECKMATE 9LA; and Atezolizumab in IMpower110, IMpower130 and IMpower150. The monoclonal antibodies used in each of these trials and/or the cytotoxic chemotherapeutic agents in each arm, the overall survival, and the hazard ratios for benefit are detailed in Table 2.

### 4.1. Keynote-024

Keynote-024 [32,33] is a phase 3 trial that compared Pembrolizumab versus platinum-based chemotherapy-doublet for PD-L1 positive NSCLC. The chemotherapy used was the investigator’s choice of platinum-based chemotherapy doublet. The trial included patients with squamous (18%) and nonsquamous (82%) histology with PD-L1 expression on at least 50% of tumor cells. Most patients were current or former smokers (92%). Median overall survival (OS) was 30.0 months with pembrolizumab and 14.2 months with chemotherapy, hazard ratio 0.63, *p* = 0.002 [32]. Pembrolizumab was associated with significantly fewer adverse events than was platinum-based chemotherapy [33]. Survival curves started to split early, about 1.5 months after the trial initiation, probably due to more efficacy and less toxicity of pembrolizumab compared to chemotherapy.

(1) Median OS in this study is 30 months, to our knowledge the longest among first line studies of NSCLC.
(2) Interestingly, females benefited much less than males with pembrolizumab compared to chemotherapy. HR for benefit among men was 0.54, and among women was 0.95. Absolute survival numbers among sexes were not published in the original [33] or updated analysis [32]. This was not a preplanned analysis, and interpretation of the results regarding the patients’ sex should be taken with caution.
(3) Never smokers had less benefit from Pembrolizumab versus chemotherapy (HR 0.9) compared to smokers (HR 0.59).

### 4.2. Keynote-042

KEYNOTE-042 [31] included NSCLC with locally advanced or metastatic disease without previous treatment and without a driving mutation in EGFR or ALK translocation, and with PD-L1 tumor proportion score (TPS) of 1% or greater. Similar to Keynote-024 [32], the trial included patients with squamous and nonsquamous histology, most of them current or former smokers [31]. This phase 3 trial compared Pembrolizumab versus the investigator’s choice of platinum-based chemotherapy doublet. Median OS was 16.7 months with Pembrolizumab and 12.1 months with chemotherapy, hazard ratio 0.81, *p* = 0.0018. For patients with PD-L1 TPS of 50% or greater, median OS was 20 and 12.2 months in the Pembrolizumab and chemotherapy groups, respectively. Interestingly, compared to Keynote-024 the survival curves opened later, at about 8 months.

(1) Compared to KEYNOTE-024, OS in KEYNOTE-042 was less even in patients with PD-L1 ≥ 50%.
(2) The similar OS in the initial months of the study between the chemotherapy and Pembrolizumab arms, trending initially to better results with chemotherapy before the curves crosses, indicates that combination therapy could provide better outcomes in a subset of patients.
(3) Female patients had less benefit compared to male patients. HR for benefit among men was 0.71 and among women was 1.01. This is consistent with KEYNOTE-024 that showed no improved survival for women with Pembrolizumab compared to chemotherapy. This was not a preplanned analysis, and interpretation of the results regarding the patients’ sex should be taken with caution.
(4) Never smokers did worse with Pembrolizumab versus chemotherapy, with HR of 1.1, compared to 0.6 and 0.71 in former and current smokers, as reported in the publication supplementary appendix [103].

### 4.3. Keynote-189 and Keynote-407

KEYNOTE-189 [104,105] and KEYNOTE-407 [103] compared chemotherapy to chemotherapy and pembrolizumab in patients with nonsquamous and squamous NSCLC, respectively, as first line therapy. There was survival benefit for pembrolizumab for patients groups with any PD-L1 expression levels [103,104]. 

#### 4.3.1. Keynote-189

This study compared chemotherapy to chemotherapy and pembrolizumab in patients with nonsquamous NSCLC as first line therapy. The chemotherapy used was Pemetrexed + Cisplatin/Carboplatin. Among patients with nonsquamous NSCLC with any PD-L1 expression level, OS was 22 versus 10.7 months with Pembrolizumab and chemotherapy compared to chemotherapy alone, HR 0.56 [104]. For patients with PD-L1≥ 50%, OS was 20.4 to not reached with Pembrolizumab and chemotherapy, compared to 10.1 months with chemotherapy alone [104]. We will be waiting for the updated analysis of this study to see if OS for combination therapy for patients with PD-L1≥ 50% will exceed those of Keynote-024 [32]. For patients with nonsquamous NSCLC and PD-L1 < 1% this trial shows clear benefit for adding Pembrolizumab to chemotherapy. Furthermore, patients with liver metastasis benefited from the combination, with OS of 12.6 versus 6.6 months in the Pembrolizumab and chemotherapy compared to chemotherapy alone groups, respectively [104]. The combination was effective in both female and male patients, and in smokers and non-smokers [105].

#### 4.3.2. Keynote-407

Keynote-407compared chemotherapy to chemotherapy and pembrolizumab in patients with squamous NSCLC as first line therapy [103]. The chemotherapy used was Carboplatin + paclitaxel or nab–paclitaxel. Among patients with squamous NSCLC with any PD-L1 expression level, OS was 15.9 versus 11.3 months with Pembrolizumab and chemotherapy compared to chemotherapy alone, respectively, HR 0.64. More than 92% of the patients were current or former smokers. Both males and females benefited from the combination [103]. Furthermore, here, OS did not reach those of Keynote-024 [32,33], and was similar to those of KEYNOTE-042 [31], which keep us with the question if there is an added benefit of chemotherapy compared to single agent Pembrolizumab in smoker male patients with PD-L1 ≥ 1%. A randomized trial in NSCLC patients, testing Pembrolizumab alone versus Pembrolizumab and chemotherapy will be needed to answer this question.

### 4.4. IMpower110

IMpower110 randomized patients with stage IV NSCLC with PD-L1 expression ≥ 1% to Atezolizumab single agent or to chemotherapy [106]. The chemotherapy used was Cisplatin or Carboplatin, combined with Gemcitabine for patient with squamous cell NSCLC, or pemetrexed for patients with nonsquamous disease. Atezolizumab was better tolerated than chemotherapy. In the subgroup of patients with EGFR and ALK wild-type tumors who had PD-L1 stained ≥ 50% of tumor cells (205 patients), the OS was 20.2 months with Atezolizumab, and 13.1 months with chemotherapy, according to the U.S. Food & Drug Administration approval and recent publication in the New England Journal of Medicine [107,108]. FDA approval is for patients with PD-L1 stained ≥ 50% of tumor cells, or PD-L1 stained tumor-infiltrating immune cells covering ≥ 10% of the tumor area, with no EGFR or ALK genomic tumor aberrations. 

### 4.5. IMpower130

IMpower130 was an open-label, phase 3 trial that compared Atezolizumab in combination with carboplatin plus nab-paclitaxel chemotherapy, with chemotherapy alone as first-line treatment for metastatic non-squamous NSCLC [56]. About half of the patients had PD-L1 negative tumors. Median OS was 18.6 months in the atezolizumab plus chemotherapy group and 13.9 months in the chemotherapy group; HR 0.79, *p* = 0.033 [56]. Subgroup analysis showed progression free survival (PFS) benefit, and a trend toward OS benefit in all PD-L1 expression levels. 

### 4.6. IMpower150

IMpower150 randomized patients with nonsquamous NSCLC to treatment with chemotherapy plus Bevacizumab, chemotherapy plus atezolizumab or chemotherapy plus Bevacizumab and atezolizumab [55,109,110]. The chemotherapy used was Carboplatin, and Paclitaxel. Median OS was 19.8 and 14.9 months for patients treated with chemotherapy plus Bevacizumab, with or without atezolizumab, respectively [55,109], (Figure 3). Median OS with Atezolizumab and chemotherapy alone was 19.5 months [109], raising question with regard to the added value of Bevacizumab to this combination for the general patients population. Importantly, patients with baseline liver metastases had an improved OS with Atezolizumab, Bevacizumab, and chemotherapy combination, compared to Bevacizumab and chemotherapy alone, with a median OS of 13.3 and 9.4 months, respectively, HR 0.52 [109]. No improvement in OS was observed for patients with liver metastasis treated with chemotherapy and atezolizumab compared to patients treated with chemotherapy and Bevacizumab [109]. Recent report about safety and patient-reported outcomes of atezolizumab plus chemotherapy and Bevacizumab shows that this drug combination seems tolerable and with manageable toxicities [110]. For patients with nonsquamous NSCLC, with baseline liver metastases, the combination of chemotherapy, Atezolizumab and Bevacizumab could be an important option to consider in the first line. 

### 4.7. Checkmate-227

**Figure 4:**
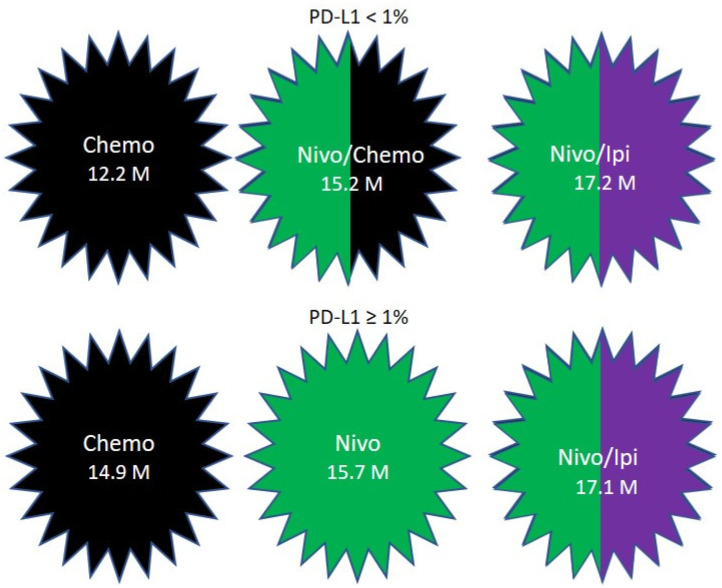
Overall survival results in CHECKMATE-227.

CHECKMATE-227 included patients with stage IV or recurrent NSCLC without previous treatment. Patients with a PD-L1 expression level of 1% or more were randomized in a 1:1:1 ratio to receive nivolumab plus ipilimumab, nivolumab alone, or chemotherapy. The chemotherapy used was Cisplatin or Carboplatin, combined with Gemcitabine for patient with squamous cell NSCLC, or pemetrexed for patients with nonsquamous disease. The OS was 17.1, 15.7 and 14.9 months, respectively [111] (Figure 4). The patients who had a PD-L1 expression level of less than 1% were randomly assigned in a 1:1:1 ratio to receive nivolumab plus ipilimumab, nivolumab plus chemotherapy, or chemotherapy. The OS was 17.2, 15.2 and 12.2 months, respectively [111] (Figure 4).

Subgroup analysis published as an appendix to the main publication [111] shows multiple interesting points:

1. Patients who never smoked had OS of 15.3 months with nivolumab plus ipilimumab compared to 16.1 months with chemotherapy alone [111].
2. Patients with PD-L1 < 1% and liver metastasis had a statistically significant benefit from nivolumab plus ipilimumab compared to chemotherapy with survival of 11.7 versus 7.8 months, respectively. This significance was not maintained in patients with PD-L1 ≥ 1 and liver metastasis. For patients, regardless of PD-L1, with liver metastasis, survival was 10.3 months with nivolumab plus ipilimumab compared to 10.4 months with chemotherapy [111].
3. Nivolumab plus ipilimumab was beneficial compared to chemotherapy in patients above and below the age of 65 years.
4. Ipilimumab was provided at a dose of 1 mg per kilogram every 6 weeks [111], which is much lower than the dose used for melanoma [112].

### 4.8. Checkmate-9LA

CHECKMATE-9LA [113] randomized patients with stage IV NSCLC, to nivolumab 360 mg Q3W + ipilimumab 1 mg/kg Q6W + two cycles of chemotherapy or 4 cycles of chemotherapy alone. The chemotherapy used was Cisplatin or Carboplatin combined with Pemetrexed or Paclitaxel. The data were presented in an abstract format and as a lecture during the American Society of Clinical Oncology (ASCO) 2020 annual meeting [113]. Median OS was 15.6 and 10.9 months, in the immunotherapy-chemotherapy and the chemotherapy only groups, respectively. Subgroup analysis showed:

(1) Clinical benefit for the combination of immunotherapy-chemotherapy was seen over chemotherapy only, regardless of PD-L1 expression.
(2) Never smokers had worse survival outcome with the immunotherapy-chemotherapy combination compared to chemotherapy only, with a median OS of 14.1 versus 17.8 months, respectively.
(3) Patients ≥ 75-year-old did worse with the immunotherapy-chemotherapy compared to chemotherapy only, with median OS of 8.5 versus 11.5 months, respectively.

## 5. Discussion

**Figure 5:**
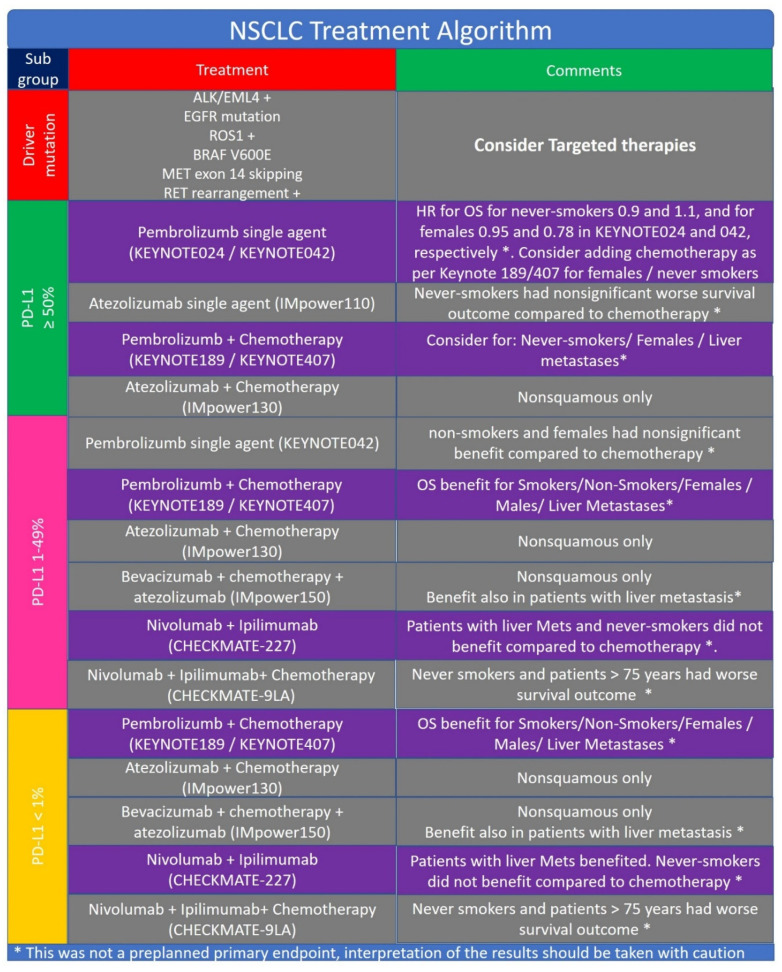
Algorithm for treatment of patients with non-small cell lung cancer (NSCLC).

The treatment of advanced/metastatic NSCLC has changed dramatically in the last 10 years. First line therapies that incorporates immunotherapy, either as a single modality or with chemotherapy, can be offered to significant part of NSCLC patients. These advances in lung cancer therapeutics, along with other causes, resulted in a decrease in population-level mortality from NSCLC in the United States from 2013 to 2016 [114]. Figure 5 provides and algorithm for treatment of patients with NSCLC.

### 5.1. PD-L1 ≥ 50%

Patients with PD-L1 ≥ 50% have multiple immunotherapy treatment options. Monotherapy is an appealing approach, with median OS of 30 months with pembrolizumab single agent compared to 14.2 months with chemotherapy, in KEYNOTE-024 [32]. OS for patients with PD-L1 ≥ 50% treated with pembrolizumab single agent in KEYNOTE-042 study was 20 months, compared to 12.2 months with chemotherapy [31]. Hazard ratio for OS benefit from pembrolizumab compared to chemotherapy for never-smokers was 0.9 and 1.1 in KEYNOTE024 and KEYNOTE042, respectively. This might indicate that never smokers do not have survival benefit with pembrolizumab compared to chemotherapy (subgroup analysis was not tested in a prospective manner). For females, HR was 0.95 and 0.78 in KEYNOTE024 and KEYNOTE042, respectively, indicating that female patients might benefit less from pembrolizumab compared to male patients, again subgroup analysis was not tested in a prospective manner. Thus for never smokers and for female patients with NSCLC we would suggest to consider adding chemotherapy to pembrolizumab as per Keynote 189 [104] and Keynote 407 [103]. In addition, finding molecular markers for aggressive, early progressing nononcogene-addicted NSCLC [115], could allow testing treatment intensification in the first line, and the role of combing immunotherapy [116] and chemotherapy in this subset of patients. 

Atezolizumab for first-line treatment of metastatic NSCLC with PD-L1 ≥ 50% was approved recently by the FDA [107], after the IMPOWER110 trial showed a median OS of 20.2 months for patients in the atezolizumab arm, compared with 13.1 months in the chemotherapy arm (*p* = 0.0106) [107,108]. 

### 5.2. PD-L1 1–49%

Monotherapy with pembrolizumab as per KEYNOTE-042 is an appealing option, especially for smoker males [31]. Combination of Nivolumab and Ipilimumab as per Checkmate 227 [111] is an option, thought there is no head to head comparison with pembrolizumab, and toxicity with the doublet is expected to be higher than in monotherapy. Pembrolizumab and chemotherapy should be considered for nonsmokers, patients with aggressive disease, and patients with liver metastases [103,104]. Atezolizumab plus chemotherapy is also an option to consider [56]. Nivolumab, ipilimumab and chemotherapy could be considered for smoker patients younger than 75 years [113]. 

### 5.3. PD-L1 <1%

Immunotherapy only (chemotherapy free) protocols suitable for this subset of patients, is a combination of Nivolumab and Ipilimumab as per Checkmate 227 [111]. Immunotherapy with 2 courses only of chemotherapy is another option for smoker patients younger than 75 years, as per Checkmate-9LA [113]. Pembrolizumab and chemotherapy could be an excellent option for many patients with PD-L1 <1% [103,104], as well as Atezolizumab plus chemotherapy [56].

### 5.4. Negative Immunotherapy Studies

While immunotherapy is now the leading treatment for many patients with lung cancer, it is important to remember that there were multiple negative trials. Durvalumab is a monoclonal antibody that block PD-L1 [74,117]. Tremelimumab is a monoclonal antibody targeting CTLA-4 [118]. Durvalumab with tremelimumab showed a manageable tolerability profile, with antitumor activity irrespective of PD-L1 status, in patients with NSCLC in a phase 1b study [119]. The combination was tested after that in phase 3 studies. The MYSTIC Trial tested Durvalumab with or without tremelimumab vs. standard chemotherapy in the first-line treatment of metastatic NSCLC [120]. The trial did not meet its primary end points of improved OS with durvalumab versus chemotherapy, or improved OS or PFS with durvalumab plus tremelimumab versus chemotherapy in patients with ≥25% of tumor cells expressing PD-L1 [120]. First-Line Nivolumab in Stage IV or recurrent NSCLC was not associated with significantly longer PFS than chemotherapy [22].

### 5.5. Chemo-Free or Chemo-Light Treatments

Until the publication of the CHECKMATE-227 and CHECKMATE 9LA studies, first line therapy for NSCLC included single agent immunotherapy with or without chemotherapy. These studies showed synergism in combining two immunotherapy medications, Nivolumab and Ipilimumab. CHECKMATE 9LA combined immunotherapy with only two cycles of chemotherapy (Chemo-light), and CHECKMATE-227 showed synergism of combining Nivolumab and Ipilimumab (Chemo-Free). Clearly these protocols could be suitable to part of the patients with low PDL-1 expression levels. The caveat for combined immune blockage with Nivolumab and Ipilimumab is the increase in immune related adverse events.

### 5.6. Durvalumab after Chemoradiotherapy in Stage III NSCLC

The PACIFIC trial tested the effect of adjuvant Durvalumab after Chemoradiotherapy in Stage III NSCLC [121,122]. PFS and OS were longer among patients who received durvalumab than among those who received placebo in patients with tumor cells with PD-L1 expression of more than 1% [121,123,124,125]. Hypofractionated thoracic radiotherapy plus Durvalumab in elderly and/or frail NSCLC stage III patients unfit for chemotherapy is currently studied in the TRADE-hypo trial [126] and the SPIRAL-RT study [127].

### 5.7. Driver Mutations

Patients with NSCLC who have a driver mutation within the tumor cells, should be considered for treatment with targeted therapies. Review of targeted therapies is beyond the scope of this article. Examples of targeted therapies include Alectinib [128]/Brigatinib [17]/Ceritinib [129]/Crizotinib [130] for patients ALK/EML4 [14,16] fusion gene. Osimertinib [131]/Erlotinib [132]/Afatinib [133]/Gefitinib [132] for patients with EGFR mutation. Crizotinib [134]/Entrectinib [135] for patients with ROS1-translocated tumors; Dabrafenib with [136,137] or without [138] Trametinib, or Vemurafenib [139] for patients with BRAF V600E mutation; Capmatinib [140] for patients with MET exon 14 skipping; and Selpercatinib for patients with RET rearrangement [141].

## 6. Conclusions

Immunotherapy is an important treatment modality for NSCLC. Tailoring the treatment to each patient is a key for achieving best benefit. While the goal should be to utilize in the first line “chemotherapy free” or “minimal chemotherapy” protocols, it is important to know which patients do not benefit from these regimens. Protocols combining immunotherapy and chemotherapy are important in the first line, especially for nonsmokers.

## Figures and Tables

**Table 1:** Antibodies targeting the immune system used for treatment of lung cancer.

Generic Name	Brand Name	Antibody Type	Indications and Usage Other than NSCLC	Target	Half-Life (Days)
Pembrolizumab	Keytruda	Humanized IgG4 kappa	Melanoma Small Cell Lung Cancer Head and Neck Squamous Cell Cancer Classical Hodgkin Lymphoma Primary Mediastinal Large B-Cell Lymphoma Urothelial Carcinoma MSI-H or dMMR Cancers Gastric Cancer Esophageal Cancer Cervical Cancer Hepatocellular Carcinoma Merkel Cell Carcinoma Renal Cell Carcinoma Endometrial Carcinoma Tumor Mutational Burden-High Cancer Cutaneous Squamous Cell Carcinoma	PD-1	22
Nivolumab	Opdivo	Fully human IgG4 kappa	Melanoma Small Cell Lung Cancer Head and Neck Squamous Cell Cancer Classical Hodgkin Lymphoma Urothelial Carcinoma MSI-H or dMMR colorectal cancer Hepatocellular Carcinoma Renal Cell Carcinoma Esophageal Squamous Cell Carcinoma	PD-1	25
Atezolizumab	Tecentriq	Humanized non-glycosylated IgG1 kappa	Urothelial Carcinoma Triple-Negative Breast Cancer Small Cell Lung Cancer Hepatocellular Carcinoma Melanoma	PD-L1	27
Ipilimumab	Yervoy	Fully human IgG1 kappa	Melanoma Renal Cell Carcinoma MSI-H or dMMR colorectal cancer Hepatocellular Carcinoma	CTLA-4	15
Durvalumab	Imfinzi	Fully human IgG1 kappa	Urothelial Carcinoma Small Cell Lung Cancer	PD-L1	18

PD-1: programmed death receptor-1; PD-L1: programmed cell death ligand 1; CTLA-4: cytotoxic T-lymphocyte antigen 4; MSI-H: Microsatellite Instability-High; dMMR: Mismatch Repair Deficient.

**Table 2:** Phase 3 clinical trials that includes Immunotherapy for NSCLC in the first line.

	Pathology	PDL-1	Arm I (OS)	Arm II (OS)	HR
KEYNOTE-024	squamous (18%) and nonsquamous (82%)	≥50%	Pembrolizumab	Investigator’s choice of platinum-based chemotherapy	
			30 months	14.2 months	0.63
KEYNOTE-042	squamous (38%) and nonsquamous (62%)	≥1%	Pembrolizumab	Investigator’s choice of platinum-based chemotherapy doublet	
			16.7 months	12.1 months	0.81
KEYNOTE-189	nonsquamous	Any level	Pembrolizumab & Pemetrexed + Cisplatin/Carboplatin	Pemetrexed + Cisplatin/Carboplatin	
			22 months	10.7 month	0.56
KEYNOTE-407	squamous	Any level	Pembrolizumab & Carboplatin + paclitaxel or nab–paclitaxel	Carboplatin + paclitaxel or nab–paclitaxel	
			15.9 months	11.3 months	0.64
CHECKMATE-227	squamous (28%) and nonsquamous (72%)	Any level ≥1% <1%	Nivolumab and Ipilimumab	Cisplatin/Carboplatin + Gemcitabine (for squamous) or pemetrexed (nonsquamous)	
			17.1 months	14.9 months	0.79
			17.2 months	12.2 months	0.62
CHECKMATE 9LA	squamous and nonsquamous	Any level	Nivolumab & Ipilimumab + Cisplatin/Carboplatin + Pemetrexed/Paclitaxel	Cisplatin/Carboplatin + Pemetrexed/Paclitaxel	
			15.6 months	10.9 months	0.66
IMpower110	squamous (25%) and nonsquamous (75%)	≥50%	Atezolizumab	Cisplatin/Carboplatin + Gemcitabine (for squamous) or pemetrexed (nonsquamous)	
			20.2 months	13.1 months	0.59
IMpower130	non-squamous	Any level	Atezolizumab & Carboplatin +nab-paclitaxel	Carboplatin +nab-paclitaxel	
			18.6 months	13.9 months	0.79
IMpower150	non-squamous	Any level	Atezolizumab + Bevacizumab + Carboplatin, and Paclitaxel	Bevacizumab + Carboplatin, and Paclitaxel	
			19.8 months	14.9 months	0.76
